# *Vibrio fluvialis*: an emerging human pathogen

**DOI:** 10.3389/fmicb.2014.00091

**Published:** 2014-03-07

**Authors:** Thandavarayan Ramamurthy, Goutam Chowdhury, Gururaja P. Pazhani, Sumio Shinoda

**Affiliations:** ^1^National Institute of Cholera and Enteric DiseasesKolkata, India; ^2^National Institute of Cholera and Enteric Diseases, Collaborative Research Center of Okayama University for Infectious Diseases in IndiaKolkata, India

**Keywords:** *V. fluvialis*, diarrhea, virulence factors, antimicrobial resistance, molecular typing

## Abstract

*Vibrio fluvialis* is a pathogen commonly found in coastal environs. Considering recent increase in numbers of diarrheal outbreaks and sporadic extraintestinal cases, *V. fluvialis* has been considered as an emerging pathogen. Though this pathogen can be easily isolated by existing culture methods, its identification is still a challenging problem due to close phenotypic resemblance either with *Vibrio cholerae* or *Aeromonas* spp. However, using molecular tools, it is easy to identify *V. fluvialis* from clinical and different environmental samples. Many putative virulence factors have been reported, but its mechanisms of pathogenesis and survival fitness in the environment are yet to be explored. This chapter covers some of the major discoveries that have been made to understand the importance of *V. fluvialis*.

## INTRODUCTION

*Vibrio fluvialis* is a halophilic Gram-negative bacterium, which has a curved cell morphology and polar flagella for motility. The important biochemical features of this organism include conversion of nitrate to nitrite, do not cleave L-lysine or ornithine, activate arginine dihydrolase, produce indole but not acetoin, ferment sucrose, D-mannitol, L-arabinose, maltose, trehalose, D-galactose, and D-galacturonate. Most of the vibrios, including *V. fluvialis* occur widely in the aquatic milieu,**mostly in the seas, estuaries and brackish waters. Even though more than 100 spices have been reported in the Genus *Vibrio* (), about 13 of them have been reported to cause several human diseases. Among the pathogenic vibrios, *V. alginolyticus, V. cholerae, V. costicola, V. mimicus, V. cincinnatiensis, V. hollisae, V. furnissii, V. parahaemolyticus, V. vulnificus, V. carchariae* (a junior synonym of *V. harveyi*) and *V. metschnikovii *are clinically important as they cause different types of vibriosis. One of the *Vibrio* spp., *V. damselae* has now been renamed as “*Photobacterium damselae* subsp. *damselae*.” The toxigenic *V. cholerae*, *V. parahaemolyticus* and *V. vulnificus* are associated with well-known cholera and diarrhea and extraintestinal infections, respectively. Prevalence of *V. cholerae* in developing countries is mostly related to the breakdown of sanitary conditions and/or due to scarcity of drinking water. On the other hand, infections caused by *V. parahaemolyticus* and other vibrios denote contamination of seafood in many countries, irrespective of their economic conditions.

*V. fluvialis* is one of the emerging foodborne pathogens all over the world. The distribution of virulence factors and molecular epidemiological features of this pathogen remain mostly unknown. Among the foodborne infections in the United States, there has been a considerable increase (43%) in the *Vibrio*-mediated infections till 2012 compared with the rates reported during 2006–2008 (Centers for Disease Control and Prevention (CDC), 2013). Several recent publications indicate the epidemiological importance of *V. fluvialis* (Chowdhury et al., 2012; Liang et al., 2013).

## IDENTIFICATION AND TAXONOMY

Thiosulfate-citrate-bile salts-sucrose agar (TCBS) has been conventionally used as a selective medium for the isolation of clinically important vibrios. The colony morphology of *V. fluvialis* in this medium remains indistinguishable from *V. cholerae,* i.e., it grows as sucrose fermenting yellow color colonies after direct plating of clinical specimens or after enrichment in alkaline peptone water (pH 8.0). After preliminary screening in the TCBS, a battery of biochemical testes is essential for the species-specific identification of *V. fluvialis*. Minimal biochemical tests such as lysine decarboxylase, ornithine decarboxylase, arginine dihydrolase, and L-arabinose are mandatory for the identification of *V. fluvialis*. Without these minimal tests, the identification is incomplete and the isolate will be improperly classified as *V. cholerae* or *Aeromonas *spp. In most resource-poor countries, these tests are not methodically performed, which may lead to labeling of *V. fluvialis* as *V. cholerae*. Considering such situation, there is a high possibility that the *V. fluvialis* could be reported as *V. cholerae *non-O1, non-O139 or non-agglutinable vibrios (NAGs). It is worth to mention here that *V. cholerae* O1 and O139 serogroups can be easily confirmed by slide agglutination with corresponding antiserum.

For the identification of *V. fluvialis* and other vibrios, rapid identification kits must be used with caution as they need additional tests for the final confirmation. While testing the commercially available identification kits, *V. fluvialis* remain as a major challenge with API 20E and Vitek GNI+ systems (Israil et al., 2003; O’Hara et al., 2003). Biochemically, *V. furnissii* expresses fibrin and mucin hydrolysis but no phosphate or esculin hydrolysis, for which *V. fluvialis* varied. *V. fluvialis*, *V. furnissii,* and *V. mimicus *are distinctive from *V. cholerae*, as the later exhibit strong mannose-sensitive hemagglutination. These test results may have a strong influence in the confirmation of strains.

Molecular tools such as PCR are useful in the identification of many uncommon vibrios and most of these assays are comparable to the conventional identification methods. The sequence of amplified 16S–23S intergenic spacers (IGSs) has demonstrated 37 ribosomal RNA (*rrn*) operons representing seven different IGS types in different *Vibrio *spp. with IGS(0), IGS(IA), and IGS(Glu) as major ones. The sequence difference in these IGS types was used to design species-specific primers for PCR for *V. fluvialis* and other vibrios (Lee et al., 2002). In some of the reports, a universal primer PCR that covers conserved regions of bacterial 16S rRNA genes followed by denaturing gradient gel electrophoresis (DGGE) was found to be useful in the identification of *V. fluvialis* either as axenic bacteria or mixed with other pathogens (Ji et al., 2004).

Initially, *V. furnissii* was taxonomically assigned with *V. fluvialis* and named as aerogenic biogroup of *V. fluvialis*. Based on DNA relatedness and several biochemical tests, *V. furnissii* has been separated as a new species (Lee et al., 1981; Brenner et al., 1983). In the phylogenetic analysis with several housekeeping genes, *V. furnissii* and *V. fluvialis* have been linked as close species. The nucleotide comparison of 16S-rRNA, *recA,* and *toxR* sequences showed that *V. furnissii* and *V. fluvialis* had 100% similarity. The gene *toxR* of *V. fluvialis* had 84% similarity with *V. harveyi* (Franco and Hedreyda, 2006). With the *gyrB*, *V. cholerae, V. mimicus,* *V. furnissii*, and *V. fluvialis* shared 93% sequence similarity.

Toxigenic vibrios have a homolog of the *toxRS* operon, which regulates the virulence expression. The gene *toxR* encodes a transcriptional activation domain (TAD), a transmembrane domain (TMD), and a periplasmic domain (PD). Among the vibrios, there is essentially no homology within the region between TAD and TMD. Hence, this region has been used in designing of primers for the species-specific identification of many vibrios. Chakraborty et al. (2006) described a species-specific identification of *V. fluvialis* by PCR targeted to the conserved transcriptional activation and variable membrane tether regions of the *toxR *gene. The functional virulence genes encoding hemolysin (*vfh*), heme-utilization (*hupO*), and central regulation (*vfpA*) have been used as targets in an multiplex PCR for the identification of *V. cholerae*, *V. parahaemolyticus*, and *V. fluvialis*, respectively (Vinothkumar et al., 2013). For the detection of clinical vibrios in seafood samples, a multiplex primer-extension reaction (PER) assay targeting the *rpoA *gene has also been reported (Dalmasso et al., 2009).

Pyrolysis-mass spectrometry with metastable atom bombardment and pattern recognition seemed to be suitable for the identification of *V. fluvialis* and other vibrios (Wilkes et al., 2005). The mass spectra have been generated via an alternative ionization method, metastable atom bombardment followed by component-discriminant analysis. Since the outer membrane protein K (OmpK) of *V. fluvialis*, *V. alginolyticus*, *V. mimicus*, *V. parahaemolyticus*, and *V. vulnificus* is highly similar, the antibodies against these proteins have been proposed in the diagnosis (Li et al., 2010). The whole cell protein profile using SDS-PAGE was also considered in the identification of clinically important vibrios including *V. fluvialis* (Lee et al., 2012).

Since simple phenotypic diagnostic tests are not available, Chen et al. (1995) used species-specific bacteriophages as a tool for the identification of *V. fluvialis* and with a set of phages, the diagnostic probability of human**isolates**was more than 84%. At least in one study, the importance of phage-typing of *V. fluvialis* has been demonstrated using six specific bacteriophages with 73% typability (Suthienkul, 1993). However, availability of these bacteriophages makes this assay technique less popular.

## PHENOTYPIC AND GENETIC CHARACTERISTICS OF *V. fluvialis*

Based on the somatic antigen variation, several serotypes of *V. fluvialis* have been identified. Though Shimada et al. (1999) identified more than 50 somatic antigens, the serological based typing of *V. fluvialis* remains non-customary. *V. fluvialis* strains belonging to serogroup O19 possessed the C (Inaba) antigen of *V. cholerae* O1, but not the B (Ogawa) or A (common) antigens (Shimada et al., 1987; Kondo et al., 2000). In the crossed immuno-electrophoresis, antibodies against the oral cholera vaccines containing killed whole cells (WC) of *V. cholerae* O1 Inaba El Tor reacted with a few strains of *V. fluvialis* (Ciznãr et al., 1989). Presence of shared WC antigens indicates that the oral cholera vaccine could stimulate immunity effectively against other vibrios also. It is known that the antigenic nature of flagella of vibrios is highly homologous. Tassin et al. (1983) and Shinoda et al. (1984) demonstrated independently that anti-L-flagella antisera of *V. fluvialis* did not agglutinate other *Vibrio *species in the H-agglutination tests. Further studies placed *V. fluvialis* and *V. furnissii* in the same lateral flagellar serogroup-HL8 (Shinoda et al., 1992). However, in practice, serotyping based on H-flagella is also not in use.

A chemotaxonomic study based on sugar composition of the polysaccharide portion of their lipopolysaccharide (LPS) has divided 35 O-antigen groups of *V. fluvialis* into 21 chemotypes (Iguchi et al., 1993). This seems to be a unique finding since the D-glycero-D-manno-heptose, and two kinds of uronic acids, i.e., galacturonic and glucuronic acids are rare in Gram-negative bacteria. In addition, 2-keto-3-deoxyoctonate, which is a typical sugar component of Gram-negative bacterial LPS was not detectable in any of the chemotypes.

Of all the molecular typing methods, the pulsed-field gel electrophoresis (PFGE) has proven to be highly useful in tying the bacterial isolates. Unlike *V. cholerae* O1 and pandemic *V. parahaemolyticu*s, the isolates of *V. fluvialis* from acute diarrheal patients exhibited large genetic diversity (Chowdhury et al., 2012, 2013).

## PREVALENCE OF *V. fluvialis* IN THE AQUATIC REALM

Even though the presence of vibrios is mostly documented from coastal environs, the domination of a particular species depends on many physico-chemical and biological factors. In warmer regions like Florida, USA, *V. fluvialis* was predominantly detected in sediments during winter months (Williams and Larock, 1985). Due to rise in seawater temperature, the identification rate of *V. fluvialis* has increased considerably (29%) in several niches at the Toulon harbor, France (Martin and Bonnefont, 1990). However, in Chesapeake Bay, *V. fluvialis* infections are always less during winter months, indirectly reflecting its minimal occurrence in this season (Hoge et al., 1989). *V. fluvialis* along with *V. vulnificus* and *V. cholerae* non-O1 unusually existed in the Seto Inland Sea of Japan, which is a eutrophic zone with riverine influence (Venkateswaran et al., 1989a). In South East Queensland, Australia, next to *V. cholerae* (10.2%), *V. fluvialis* (8.2%) has been isolated more frequently from river waters, sediments, and plants (Myatt and Davis, 1989).

Due to high load of pollution in the upstream of the river Ganges, presence of *V. fluvialis* (0.74%) with other potential pathogens have been detected in several points of Varanasi, India (De et al., 1993). *V. fluvialis* has also been isolated from natural waters in Myanmar (Oo et al., 1993) and in a wide range of coastal environments of Japan (Uchiyama, 2000). Compared to other vibrios, the recovery of *V. fluvialis* has been high (41.4%) from suburban community effluents in South Africa. However, their occurrence was not associated with any season or plankton blooms, but positively correlated with temperature, salinity, and dissolved oxygen (Igbinosa et al., 2011a).

In many investigations, the detection frequency of *V. fluvialis* was very high in marine mollusks, mostly in bivalves, as they accumulate large number of pathogens during the process of filter-feeding. Findings of Kelly and Stroh (1988) from Pacific Northwest showed that oysters are the main source of *V. fluvialis* and other vibrios especially during warmer seasons. In Hong Kong, *V. fluvialis* was one of the important pathogenic vibrios identified in coastal waters and seafood sold in the markets (Chan et al., 1986, 1989). *V. fluvialis* has been isolated from mussels from Senegal (Schandevyl et al., 1984), Brazil (Matté et al., 1994), bivalves and mud from Costa Rica (García and Antillón, 1990) and cultured fishes from Denmark (Pedersen et al., 1999), copepods from Southern Italy (Dumontet et al., 2000) and cockles of Malaysia (Elhadi et al., 2004).**In Turkey, next to *V. alginolyticus *(>30%), *V. fluvialis* was the most common *Vibrio *in blue crabs and retail fishes (>10%; Yalcinkaya et al., 2003; Yücel and Balci, 2010).

Generally, fecal pollution has been monitored in aquaculture areas to forecast human pathogens in the products. In Italy, about 11–27% of the mollusks and shrimps contained *V. fluvialis* without any association between presence of this pathogen and conventional fecal pollution indicators (Ripabelli et al., 2004). The micro fauna and flora occasionally support the occurrence human pathogens. *V. fluvialis* (36.5%) was significantly associated with plankton in the effluents of a rural wastewater treatment facility in the Eastern Cape Province of South Africa (Igbinosa et al., 2009). In the Atlantic coast of France, Deter et al. (2010) showed that chlorophyll-A had a significant influence on pathogenic vibrios including *V. fluvialis* in mussels.

There are few reports about identification of *V. fluvialis *from wound infections that took place in recreational areas. Since *V. fluvialis *has been**cultured from the teeth of a great white shark**(*Carcharodon carcharias*),**there may be an association of this pathogen with wound infections caused by sharks in humans (Buck et al., 1984). Fibropapillomatosis (FP) is a mutilating disease among turtles that cause tumors on the skin and other internal organs. In a study conducted by Aguirre et al. (1994) showed the presence of *V. fluvialis* (47%) in green turtles with FP.

In the marine environment, *V. fluvialis *plays a major role in the production of hydrogen from starch acquired from the algal mass in the presence of *Rhodobium marinum*. In co-culture experiments, *V. fluvialis* degrade starch leading to the formation of acetic acid and ethanol, which are subsequently utilized for hydrogen production by *R. marinum *(Ike et al., 1999).

## SPORADIC CASES AND OUTBREAKS OF DIARRHEA DUE TO *V. fluvialis*

Early reports from the US indicated involvement of *V. fluvialis* with gastroenteritis among infants (Hickman-Brenner et al., 1984; Bellet et al., 1989; Kolb et al., 1997). Since 1979, *V. fluvialis* was isolated as one of the important pathogens in Tenri Hospital, Japan (Aihara et al., 1991). Prevalence of *V. fluvialis* among children with diarrhea was very less during 1988 (0.6%) in Calcutta (now, Kolkata), India (Chatterjee et al., 1989). In the same region, progressive increase in the prevalence of *V. fluvialis* (>2%) among hospitalized acute diarrheal patients has been reported in the following years (Chowdhury et al., 2012). During 1996–1998, prevalence of *V. fluvialis* was 9.4% among hospitalized diarrheal patients in North Jakarta (Lesmana et al., 2002). In Zhejiang Province, China, *V. fluvialis* was identified as the second most pathogen (12%) among acute diarrheal cases but next to *V. parahaemolyticus* (64%; Jiang, 1991). Investigations carried out after the 1998 floods in Bangladesh showed involvement of *V. fluvialis* in a diarrhea outbreak (Tanabe et al., 1999). However, the number of cases was less compared to *V. cholerae* O1 and O139 infections.

*Vibrio-*mediated infections frequently occur in countries where the raw seafood is largely consumed. In many instances, *V. fluvialis *was**found to be associated with cholera-like diarrhea (Allton et al., 2006). Between 1982 and 1988, 10 gastroenteritis cases of *V. fluvialis* have been reported in Florida due to consumption of contaminated seafood (Klontz and Desenclos, 1990). In the Gulf coast, the majority of the *Vibrio*-mediated gastroenteritis has been associated with intake of raw oysters and in about 6% of the cases *V. fluvialis* was the causative pathogen (Levine and Griffin, 1993). Foodborne outbreaks were reported in several communities implicating *V. fluvialis* alone or with either *V. parahaemolyticus/Salmonella* spp. (Tokoro et al., 1984; Chowdhury et al., 2013).

Foodborne diarrheal outbreaks caused by *V. fluvialis* have been reported during 1981 in Maharashtra (Thekdi et al., 1990) and 2012 in Kolkata (Chowdhury et al., 2013). In Brazil, and USSR, the first report on the association of *V. fluvialis* with diarrhea was reported during 1990 and 1991, respectively ((Magalhães et al., 1990; Libinzon et al., 1991). Though the incidence of cholera among high socioeconomic population in Brazil was very low (0.07%), but the other vibrios including *V. fluvialis* comparatively prevailed more (1.2%; Magalh a, 1993). In Volga delta, Russia, acute enteric infections caused by *V. fluvialis* reaches about 30% during the summer months, mainly due to consumption of water than sea/fresh water fishes (Boui, 2000). Among travelers with diarrheal symptoms, the incidence of *V. fluvialis* seems to be low compared to other enteric pathogens. Early studies conducted with US Peace Corps volunteers in Thailand identified *V. fluvialis* in about 3% of the cases (Taylor et al., 1985).

## OTHER INFECTIONS

*Vibrio fluvialis* causes a variety of infections in immune-competent/HIV patients, including bacteremia, biliary tract infection and acute diarrhea (Albert et al., 1991; Usó et al., 2010; Liu et al., 2011). The other rarely reported infections caused by this pathogen include suppurative cholangitis (Yoshii et al., 1987), peritonitis (Lee et al., 2008), acute otitis (Cabrera et al., 2005; Chen et al., 2012) and endophthalmitis (Penland et al., 2000). Large numbers of (29%) endophthalmitis patients were reported to have mixed infection with *V. fluvialis* (Hassan et al., 1992). A report from Cuba showed that *V. fluvialis* was one of the predominantly identified pathogens from different extraintestinal samples (Cabrera et al., 2007). Cases of bacteremia with diarrhea (Lai et al., 2006) hemorrhagic cellulitis and cerebritis (Huang and Hsu, 2005), peritonitis (Ratnaraja et al., 2005) have also been reported.

## QUORUM SENSING

Quorum sensing (QS) is a process in which bacterial cells in a population are able to crosstalk with one another, thereby supporting them as a unit to synchronize gene regulation and consequent phenotypic changes. The importance of QS in pathogenic *V. cholerae* has been well established. Wang et al. (2013) have shown that QS in *V. fluvialis* regulates two potential virulence factors, including an extracellular protease and hemolysin. In addition, QS also regulates *in vitro* cytotoxic activity against epithelial cell lines.

## VIRULENCE FACTORS

The clinical as well as environmental *V. fluvialis* strains express many putative virulence factors. The common virulence factor in *V. fluvialis* reported in several investigations is the expression of hemolysin that can be easily identified in sheep-blood agar plates. In majority of the toxin detection assays, eukaryotic cell lines are being used* in vitro*. In cell-free extracts, *V. fluvialis* has expressed Chinese hamster ovary (CHO) cell elongation factor, CHO cell-killing factors, cytolysins against erythrocytes and proteases active against azocasein (Lockwood et al., 1982). Various putative virulence factors of *V. fluvialis* are presented in **Table 1**. However, the ability to produce these factors is not uniform in all the isolates (Liang et al., 2013).

**Table 1:** Different putative virulence factors described in *V. fluvialis*.

Factor	Reference
Cytolysin	Lockwood etal. (1982)
Heat-labile cytotoxin	Wall etal. (1984)
Cytotonic	Venkateswaran etal. (1989b)
Hemolysin	Wong etal. (1992)
Mucinase	Janda (1986)
Mannose sensitive hemaagglutination	Rahman etal. (1992)
Cell adherence	Carvalho etal. (1994), Scoglio etal. (2001)
Cell vaculation	Chakraborty etal. (2005)

Purification of cytotoxin produced by *V. fluvialis* showed that the protein was heat-labile, and deactivated by proteases. The culture supernatant retained hemolytic and phospholipase A2 activities and were coeluted in the gel filtration (Wall et al., 1984). The purified extracellular hemolysin produced by *V. fluvialis* showed virulence features including lyses of erythrocytes of different animal origin and activation of fluid accumulation in suckling mice (Han et al., 2002; Kothary et al., 2003).

The transmembrane regulatory protein (ToxR) is essential for the expression of virulence factors in pathogenic vibrios. Similar to *V. cholerae*, the ToxR plays a major role in bile resistance of *V. fluvialis*, which is an initial phase in the progression of vibrios as potential intestinal pathogens (Provenzano et al., 2000). Adaptability of vibrios to the intestinal environment, especially the bile salts favors colonization and expression of virulence factors. After initial adaptation to the bile salts under *in vitro* conditions, the *V. fluvialis* exhibited swarming mobility, biofilm formation and adherence (Di Pietro et al., 2004). In the animal models, *V. fluvialis *and the cholera toxin (CT) produced by *V. cholerae* O1 strains confirmed skin permeability factor (SPF). However, the antibodies against CT did not neutralize the SPF of *V. fluvialis* (Rodrigues et al., 1993; Ahsan et al., 1988).

The exocellular metalloprotease produced by *V. fluvialis* (VFP) was found to be similar to the one produced by *V. vulnificus*, which has also been used for the hemagglutination activity (Miyoshi et al., 2002). In addition, the amino acid sequence of VFP was found to be a member of the thermolysin family. It is interesting to note that most of the *V. fluvialis* isolated from the diarrheal patients harbored genes encoding hemolysin and metalloprotease (Chowdhury et al., 2012).

## SURVIVAL

*Vibrio fluvialis *has the capacity to survive in the seawater microcosm for more than 15 days at ambient temperature regardless of carbonated substrate uptake (Munro et al., 1994). In microcosms, *V. fluvialis* has been shown culturally viable for a year without losing its virulence and in sediments this organism was recovered from viable but non-culturable stage, even after 6 years (Amel et al., 2008).

## ANTIMICROBIAL RESISTANCE

Compared to other clinical vibrios, antimicrobial resistance (AMR) is largely reported in *V. fluvialis*. In Mediterranean fish farms, many of the vibrios including *V. fluvialis* were resistant to ampicillin, carbenicillin, kanamycin, cefalotin, and sulfadiazine-trimethoprim (Laganà et al., 2011). In South Africa, treated effluent system was found to be the reservoir for *V. fluvialis* strains, which are resistant to ampicillin, penicillin-G, streptomycin, sulfamethoxazole, trimethoprim, chloramphenicol, erythromycin, ciprofloxacin, and polymyxin B (Igbinosa et al., 2011b). In China, majority of the *V. fluvialis* strains were resistant for β-lactams, azithromycin, and sulfamethoxazole (Liang et al., 2013).

Several mobile genetic elements carrying AMR have been found in *V. fluvialis. *The integrative and conjugative element (ICE) is a conjugative transposon commonly detected in *V. cholerae*, which carries resistance genes for sulfamethoxazole-trimethoprim (SXT), chloramphenicol and streptomycin (Srinivasan et al., 2006; Taviani et al., 2008). This SXT element has also been reported in *V. fluvialis* that has integrase gene similar to that of *V. cholerae* (Ahmed et al., 2005). The aminoglycoside acetyltransferase encoding gene *aac(3)-Id* was identified in class 1 integron from a clinical *V. fluvialis* strains (Ahmed et al., 2004).

Transfer of large plasmids carrying AMR genes is rarely detected in *V. fluvialis* (Rajpara et al., 2009). Efflux systems responsible for nalidixic acid and ciprofloxacin resistance have been reported in several clinical *V. fluvialis* strains (Srinivasan et al., 2006). Two putative multi antimicrobial extrusion (MATE) protein family efflux pumps viz., H- and D-type were found to be responsible for fluoroquinolones resistance in *V. fluvialis*. The sequences of these MATE encoding genes were found to be ~99% identical to *V. cholerae* (Mohanty et al., 2012). In addition, many *V. fluvialis* strains had mutation (serine to isoleucine) at position 83 of the quinolone resistance-determining region (QRDR) of *gyrA*. Apart from this mutation, presence of plasmid-borne *qnrVC*-like genes have been reported for quinolone resistance in some of the *V. fluvialis* strains (Singh et al., 2012). *V. fluvialis* isolated from diarrheal patients in Kolkata were resistant to fluoroquinolones and β-lactam antimicrobials had mutations in the QRDR of GyrA at position 83 and of ParC at position 85 (Chowdhury et al., 2011). In addition, these strains carried a transferrable 150-kb plasmid that harbored the quinolone resistance *qnrA1* in a complex *sul1*-type integron, the ciprofloxacin-modifying enzyme-encoding gene *aac(6′)-Ib-cr* and genes encoding for extended-spectrum β-lactamases such as *bla_SHV_* and *bla_CTX-M-3_*.

## CONCLUSION

Though the pathogen *V. fluvialis* has known for quite some time, its clinical importance is realized now, as the prevalence of diarrhea cases is reportedly increasing. In depth studies on the pathogenesis of *V. fluvialis* has to be established as there are many descriptions about the putative virulence factor.

## Conflict of Interest Statement

The authors declare that the research was conducted in the absence of any commercial or financial relationships that could be construed as a potential conflict of interest.
